# Sociodemographic Determinants of Psychological Distress Among Adult Informal Caregivers

**DOI:** 10.7759/cureus.64154

**Published:** 2024-07-09

**Authors:** Clifford Atuiri, Emmanuel Afful, Ruth Zeto, Isaac Che, Xiao Li, Enoch Azasu

**Affiliations:** 1 Internal Medicine, St Luke's Hospital, St. Louis, USA; 2 Brown School, Washington University in St. Louis, St. Louis, USA; 3 Public Health, Harvard School of Public Health, Boston, USA; 4 Psychiatry, Saint Louis University School of Medicine, St. Louis, USA

**Keywords:** informal caregivers, mental health, family caregivers, demographic, caregiver, psychological distress

## Abstract

Background and objective

Providing care for a loved one with a chronic illness or disability can be mentally and emotionally challenging. Determining the factors that contribute to psychological distress among informal caregivers can be important in developing effective interventions to support this vulnerable population. This study aimed to examine the sociodemographic determinants of psychological distress among adult informal caregivers in the United States.

Research design and method

Secondary data analysis using the 2022 Health Information National Trends Survey was conducted. A total of 807 informal caregivers were included in the study. Sociodemographic characteristics, caregiving conditions, and caregivers' relationship to care recipients were assessed. Psychological distress was measured using Patient Health Questionnaire 4. Weighted multivariate logistic regression analysis was conducted to determine the associations between sociodemographic factors and psychological distress.

Results

The prevalence of psychological distress was 40%. The average age of the sample was 56 years with most caregivers being female and non-Hispanic White. Older age was associated with lower odds of distress (OR=0.974, 95% CI: 0.949-0.999). Female caregivers had higher odds of distress compared to males (OR=1.922, 95% CI: 1.023-3.612), and caregivers with household incomes of $75,000 or more had significantly lower odds of distress compared to those with incomes below $35,000 (OR=0.266, 95% CI: 0.119-0.595). Race/ethnicity and educational level did not show significant associations with caregiver distress.

Conclusion

Younger age, female birth gender, and lower household income were associated with higher odds of distress among informal caregivers. These findings can inform the development of targeted interventions to support caregivers’ mental health.

## Introduction

Informal caregivers constitute a substantial and indispensable part of the U.S. healthcare system, providing essential support to individuals with chronic conditions and disabilities [1]. According to the National Alliance for Caregiving, there has been an over 9.5 million increase in the number of caregivers in the U.S. from 2015 to 2020, with one in five Americans considered caregivers [2]. The number of informal caregivers in the U.S. has been estimated to increase over the next decades, due to the increasing number of aged population in the country [3]. About 80% of adults requiring long-term care are currently living at home, with informal caregivers providing about 90% of their care [4].

Informal caregiving can be a source of physical, emotional, and financial support to care recipients. However, the responsibilities and challenges faced by informal caregivers can have profound implications for their well-being, particularly their mental health [5]. Psychological distress, including symptoms of depression, anxiety, and emotional exhaustion, can significantly impact the mental health and quality of life of informal caregivers, ultimately affecting the quality of care provided to the care recipients [6].

While numerous studies have examined the psychological distress experienced by informal caregivers, a comprehensive analysis of the sociodemographic determinants of psychological distress among informal caregivers using a large sample size is lacking. Also, most of the studies focused on caregivers visiting health centers, limiting the generalizability of these studies. Recognizing the multidimensional nature of caregiving and exploring how sociodemographic factors intersect and influence caregiver well-being is imperative. Such an understanding will aid in identifying vulnerable caregiver populations and developing tailored interventions to mitigate psychological distress and improve overall caregiver well-being.

This study aims to contribute to the existing literature by examining various sociodemographic factors that influence psychological distress among informal caregivers by using a large and inclusive study. By focusing on age, birth gender, ethnicity, household income, educational level, relationship to the care recipient, and caregiving condition, we aim to characterize the diverse variables that shape a caregiver’s well-being. This study seeks to illuminate the complex interplay of these factors, which will contribute to the development of evidence-based strategies for supporting caregivers and improving their mental health outcomes.

The outcomes of this research have important implications for policy development and healthcare planning. Ultimately, by prioritizing the well-being of informal caregivers, we can enhance the quality of care provided to vulnerable individuals, promote sustainable caregiving practices, and improve the overall functioning of the healthcare system.

## Materials and methods

Data and study population

This study was conducted using data from the National Cancer Institute’s Health Information National Trend Survey (HINTS). HINTS is a publicly available, nationally representative cross-sectional survey about the American public’s knowledge, attitudes, and use of health-related information. The survey included only adults aged 18 or older in a civilian non-institutionalized setting in the United States. The HINTS 6 (2022) dataset was used. More information about HINTS has been previously described in detail [7].

Informal caregivers were the population of interest. Participants were asked in the survey “Are you currently caring for or making health care decisions for someone with a medical, behavioral, disability, or other condition?” Those who answered “Yes” to this question were classified as caregivers and those who selected “No” to the question were excluded from the analysis.

**Figure 1:**
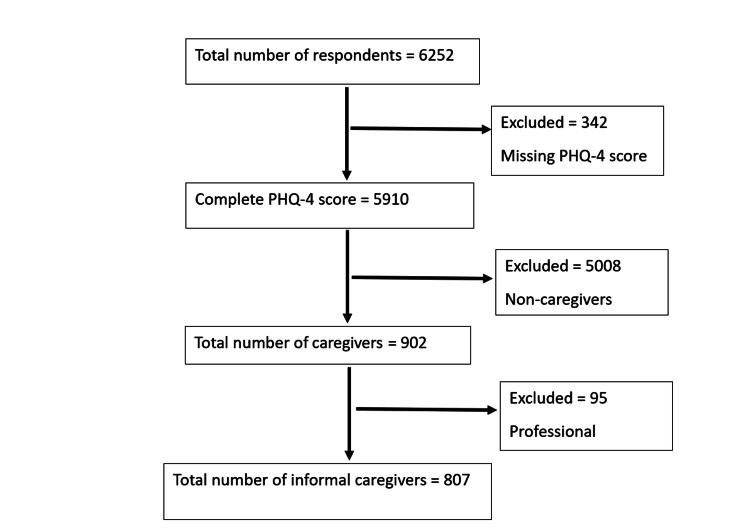
Flowchart of final sample size for analysis

Respondents were further asked, “Do you provide any of this care professionally as part of a job (for example, as a nurse or professional home health aide)?”. Those who selected “No” to this question were classified as “Informal Caregivers”. Participants who answered “Yes” to providing care as a professional service were excluded from the analysis. A flowchart of the final sample size is displayed in Figure 1.

Study measures

The outcome of interest was psychological distress, which was operationally defined using the Patient Health Questionnaire-4 (PHQ-4). The PHQ-4 is a validated and standardized brief screening tool for anxiety and depression [8]. Participants were classified as normal (PHQ-4 score <3) or under psychological distress (PHQ-4 score >=3) depending on their total PHQ-4 Score. Participants with missing data for the PHQ-4 variable were excluded from the analysis.

Age, birth gender, race/ethnicity, educational status, household income, and educational level were the predictors included in the analysis. Age was included in the analysis as a continuous variable. For birth gender, participants were asked “On your original birth certificate, were you listed as male or female?”. The birth gender categories were maintained for the analysis. All the other predictors were included as categorical variables in the analysis. The race/ethnicity categories in the survey were Non-Hispanic White, Non-Hispanic Black or African American, Hispanic, Non-Hispanic Asian, and Non-Hispanic Other. The Hispanic, Non-Hispanic Asian, and Non-Hispanic Other groups were combined into “Others” for the analysis due to the small sample size in each category. Household income was re-categorized as less than $35,000, $35,000 to less than $75,000, and $75,000 or more. The educational status categories were High school or less, Some college, and College degree or more. All other categories of predictors were maintained from the survey.

Data analysis

Descriptive statistics were computed to summarize the sociodemographic characteristics of informal caregivers. Continuous variables were summarized with means and standard deviations while categorical variables were summarized as counts and percentages. Chi-square tests were used to assess differences in sociodemographic categories among those with and without psychological distress and an independent t-test was used for continuous variables.

To explore the unique associations of sociodemographic factors on caregivers’ distress, a weighted multivariate logistic regression analysis was performed to estimate the association between sociodemographic characteristics and caregivers' distress. The care recipients’ condition was significantly associated with caregivers’ psychological distress in a bivariate analysis and hence was adjusted for in the multivariate model. The various assumptions of logistic regression were assessed to ensure the robustness of the multivariate model. The Box Tidwell test was used to test the linearity between continuous sociodemographic predictors and psychological distress. The age of respondents was a continuous variable, and it met the assumption of linearity. All the other independent variables were categorical. Cook’s distances for the observations were calculated to identify outliers and overly influential points in the sample. There were no outliers or overly influential observations in the sample. The variance inflation factor (VIF) was used to assess multicollinearity, which showed no multicollinearity between the independent variables. Jackknife weighting was employed in the regression analysis. The rationale behind using the jackknife weighting technique in this study was to mitigate potential biases arising from sample selection and non-response. It allowed for the estimation of population parameters and standard errors while accounting for the complex sampling design in HINTS and potential dependence among observations. The technique provides more robust and representative estimates by reducing the impact of outliers and influential observations.

Ethical considerations

The HINTS survey adheres to strict ethical guidelines and maintains the confidentiality of participants' information. The survey was conducted with informed consent from participants, and all data collected were de-identified to ensure privacy and compliance with ethical standards. The present study obtained access to the HINTS dataset following the necessary data request and approval process, adhering to the ethical guidelines and terms of use set forth by the National Cancer Institute. Our study follows the reporting guidelines of the Strengthening the Reporting of Observational Studies in Epidemiology (STROBE).

## Results

Participant characteristics

The total unweighted sample for the analysis was 807, which translates to a weighted estimate of 37,256,510. Table 1 displays unweighted participants’ sociodemographic characteristics. Most of the informal caregivers were female (68%), non-Hispanic White (54%), and between the ages of 50 and 64 years (36%). Most of the caregivers had at least a college degree (49%) and a household income of $75,000 or more (41%). The prevalence of psychological distress in the sample was 40%. There was a significant difference in who did and did not have psychological distress by age, household income, and education.

**Table 1:** Unweighted descriptive statistics of informal caregivers Note: Not every total adds up to 807. Missing responses to demographic questions were excluded. S.D = Standard Deviation

Variables	Total N=807	No psychological distress N=488	Psychological distress N=319	p-value
	N (%)	N (%)	N (%)	
Age				<0.001
Age in years	Mean=56 S.D = 15	Mean= 58 S.D =15	Mean=54 S.D = 16	
Birth gender				0.25
Male	252 (32%)	158 (34%)	94 (30%)	
Female	528 (68%)	308 (66%)	220 (70%)	
Race/ethnicity				0.11
Non-Hispanic White	402 (54%)	253 (57%)	149 (49%)	
Non-Hispanic Black	130 (17%)	70 (16%)	60 (20%)	
Others	215 (29%)	122 (27%)	93 (31%)	
Household income				<0.001
Less than $35,000	194 (27%)	83 (19%)	111 (37%)	
$35,000 to	241 (33%)	133 (31%)	108 (36%)	
$75,000 or more	297 (41%)	214 (50%)	83 (27%)	
Education				0.046
High school or less	157 (20%)	83 (18%)	74 (24%)	
Some college	242 (31%)	140 (30%)	102 (32%)	
College graduate or more	382 (49%)	244 (52%)	138 (44%)	

Table 2 displays caregiving conditions and the relationship between caregivers and care recipients. Most of the caregivers offered care to recipients with multiple conditions (40%). This was followed by caregivers of recipients with mental health issues, substance abuse, and intellectual or developmental issues only (12%). There were significant differences in psychological distress by the type of caregiving condition. Most of the respondents offered care to a parent (27%). There was no significant difference in psychological distress between the various relations of caregivers to care recipients.

**Table 2:** Caregiving characteristics

Characteristic	Total	No psychological distress	Psychological distress	p-value
	N=807	N=488	N=319	
Relationship to care recipient				0.22
Spouse/Partner only	166 (21%)	98 (20%)	68 (21%)	
Parent(s) only	216 (27%)	140 (29%)	76 (24%)	
Friend/Other non-relative only	26 (3%)	18 (4%)	8 (3%)	
Multiple caregiving relationships	99 (12%)	51 (10%)	48 (15%)	
Another family member only	111 (14%)	63 (13%)	48 (15%)	
Child/Children that need(s) special care due to a medical condition or disability only	189 (23%)	118 (24%)	71 (22%)	
Caregiving condition				0.003
Cancer only	22 (3%)	15 (3%)	7 (2%)	
Not sure/Don't know	44 (6%)	27 (6%)	17 (5%)	
Multiple caregiving conditions	309 (40%)	169 (36%)	140 (45%)	
Alzheimer's, confusion, dementia, forgetfulness, brain injury, stroke, or other neurological issue only	90 (12%)	66 (14%)	24 (8%)	
A short-term but serious condition such as recovery from surgery or an injury only	16 (2%)	13 (3%)	3 (1%)	
A long-term illness such as high blood pressure, hypertension, diabetes, heart disease, heart attack, lung disease, or emphysema only	73 (9%)	40 (8%)	33 (11%)	
Difficulty moving around such as an orthopedic issue, a musculoskeletal issue, or an aging-related issue only	55 (7%)	43 (9%)	12 (4%)	
A mental health issue, substance abuse, intellectual or developmental issue only	117 (15%)	65 (14%)	52 (17%)	
Other only	56 (7%)	33 (7%)	23 (7%)	

Sociodemographic determinants of psychological distress

Table 3 shows the weighted adjusted odd ratios of psychological distress by sociodemographic characteristics. Caregiver’s age was a significant predictor of psychological distress. For every 1-year increase in age, there were 2.6% lower odds of psychological distress (OR= 0.974 95% CI: 0.949 - 0.999, p<0.05). Female caregivers had 92% higher odds of psychological distress than male caregivers. This difference in odds between males and females was statistically significant (OR= 1.922, 95% CI: 1.023 - 3.612, p<0.05).

**Table 3:** Weighted multivariate logistic regression of the association between sociodemographic predictors and psychological distress ^a^ adjusted for care recipients' condition and all other variables in the multivariate model

	Adjusted OR (95% CI)^a^	P-value
Age	0.974 (0.949 – 0.999)	0.046
Birth gender		
Male	Ref.	
Female	1.922 (1.023 - 3.612)	0.043
Race/Ethnicity		
Non-Hispanic White	Ref.	
Non-Hispanic Black	1.243 (0.625 - 2.473)	0.528
Others	1.492 (0.602 – 3.701)	0.380
Household income		
Less than $35,000	Ref.	
$35,000 to	0.768 (0.360 - 1.635)	0.485
$75,000 or More	0.266 (0.119 - 0.595)	0.002
Education		
High school or less	Ref.	
Some college	1.438 (0.619 – 3.343)	0.391
College degree or more	1.329 (0.615 - 2.872)	0.461

Caregivers who identified as non-Hispanic Black or African American had 24.3% higher odds of psychological distress (OR= 1.243, 95% CI: 0.625 - 2.473, p>0.05), and other race/ethnicity groups had 49.2% higher odds of psychological distress (OR= 1.492, 95% CI: 0.602 - 3.701, p>0.05) when compared with their non-Hispanic White counterparts. However, the differences between the racial groups were not statistically significant. The odds of psychological distress among caregivers with household incomes in the range of $35,000 to $75,000 was 23.2% lower than caregivers with a household income below $35,000. However, this difference was not statistically significant (OR=0.768, 95% CI: 0.360 - 1.635, p>0.05). Caregivers with a household income of $75,000 or more had significantly lower odds (73.4%) of psychological distress than caregivers with a household income of less than $35,000 (OR=0.266, 95% CI: 0.119 - 0.595, p<0.05).

Caregivers with some college education and those with a college degree and more had higher odds of psychological distress than those with a high school education or less. However, these were not statistically significant (OR=1.438, 95% CI: 0.360 - 1.635, p>0.05 and OR=1.329 95% CI: 0.615 - 2.872, p>0.05 for some college education and college degree or more respectively).

## Discussion

This study aimed to investigate the sociodemographic determinants of psychological distress among informal caregivers. The findings revealed several significant associations between certain socio-demographic factors and psychological distress, providing valuable insights into the factors influencing caregivers’ well-being.

Age emerged as a significant predictor of psychological distress among caregivers. The results indicated that older age was protective against psychological distress. This finding suggests that younger caregivers may face additional challenges and stressors in their caregiving role, which may contribute to higher levels of psychological distress. Previous studies in caregiver subgroups have shown a similar trend of higher psychological distress among younger caregivers [9,10]. This observation may be attributed to older caregivers having more life experience, greater emotional resilience, and better coping mechanisms. Additionally, older caregivers may have developed stronger social support networks and access to resources, which can buffer the impact of caregiving stress. Interventions targeting younger caregivers should consider their unique needs and focus on providing education about caregiving responsibilities, stress management techniques, and support in balancing caregiving with other life domains such as education and career development.

Gender differences in psychological distress among caregivers were also evident in our study, with female caregivers exhibiting higher odds of psychological distress compared to their male counterparts. This aligns with previous research highlighting the gendered nature of caregiving and the differential impact on mental health outcomes [11]. Caregiving roles may impose additional burdens on women, who often shoulder multiple caregiving responsibilities alongside other familial and societal roles [12,13]. Also, societal expectations, gender roles, and the double-duty responsibilities often assumed by female caregivers may contribute to their elevated distress levels. Gender-sensitive interventions, including support groups, addressing societal norms, counseling services, and stress management programs, can help address the specific needs of female caregivers and alleviate their distress [14].

While there were no statistically significant differences in psychological distress between racial and ethnic groups in our study, certain trends were observed. Caregivers identifying as non-Hispanic Black or African American and other race/ethnicity groups tended to have higher odds of psychological distress compared to non-Hispanic White caregivers. Although these differences were not statistically significant, it is essential to recognize the potential influence of racial and ethnic factors on caregiver distress. Cultural differences, discrimination, and disparities in healthcare access and resources may contribute to the elevated distress levels observed among certain racial and ethnic groups. Findings from studies assessing the race/ethnic difference in mental health in informal caregivers have shown varying results for the association between race/ethnicity and psychological distress [15,16]. Further studies involving a more diverse population are needed to elucidate the mental health impact of caregiving on different race/ethnic groups and the potential mediators of this association. Considering the potential influence of race and ethnicity on caregiver distress, culturally sensitive interventions should be developed to address the specific needs and stressors experienced by caregivers from different racial and ethnic backgrounds. This may involve providing culturally appropriate information, and language services, and connecting caregivers with community resources that are sensitive to their cultural values and beliefs.

Socioeconomic factors, specifically household income, demonstrated significant associations with psychological distress. Caregivers with a household income of $75,000 or more had significantly lower odds of distress compared to those with a household income below $35,000. Financial resources and stability may contribute to better access to healthcare services, respite care, and support networks, thereby reducing caregiver distress. Lower-income caregivers may face additional financial strain, limited access to support services, and reduced opportunities for self-care, contributing to their higher distress levels. Financial strain is a known predictor of mental health in the general population, and higher household income may provide greater access to resources, healthcare services, and support networks, reducing the burden of distress [17]. Strategies to alleviate financial strain and increase access to support services for lower-income caregivers can help mitigate the impact of financial barriers on their well-being. This may include providing financial assistance, facilitating access to affordable respite care, and connecting caregivers with community resources for financial support.

Education level showed a trend toward higher odds of psychological distress among caregivers with some college education or a college degree compared to those with a high school education or less. Although not statistically significant, this finding suggests that higher education may not necessarily protect against caregiver distress. Education can be a double-edged sword, as caregivers with higher education may possess more awareness of the challenges and responsibilities of caregiving, leading to heightened distress. Additional research is needed to explore the potential mediating factors, such as caregiver self-efficacy, coping mechanisms, and social support networks, which may influence the relationship between education and psychological distress.

Overall, the findings of this study highlight the importance of adopting a multidimensional and tailored approach to address caregiver distress. Interventions should consider the intersectionality of sociodemographic factors and develop strategies that are sensitive to the unique needs and challenges faced by caregivers from diverse backgrounds. Collaborative efforts among healthcare providers, policymakers, and support organizations are necessary to implement and evaluate the effectiveness of these interventions.

This study possesses several strengths that enhance the validity and generalizability of its findings. The study utilized a relatively large nationally representative sample of informal caregivers, providing a robust representation of the caregiver population. Also, the study utilized the jackknife weighting technique, a robust statistical method, to address potential bias due to non-response and selection issues. The use of the jackknife weighting technique strengthens the reliability of the results and enhances the methodological rigor of the study. The study employed adjusted odds ratios to account for potential confounding variables, ensuring that the observed associations between sociodemographic factors and caregiver distress were not solely influenced by other factors. Adjusting for potential confounders strengthens the validity of the findings and enhances the accuracy of the estimated effects of sociodemographic determinants on caregiver distress.

It is worth noting some limitations of this study. First, the study relied on self-reported measures of psychological distress, which may be subject to reporting biases. Future studies could employ objective assessments of distress to enhance the validity of the findings. Second, the study focused on sociodemographic determinants and did not explore other potential factors that may influence caregiver distress such as social support, caregiver burden, and care recipient characteristics. Future research should consider incorporating these variables to provide a more comprehensive understanding of caregiver distress. Lastly, the study utilized cross-sectional data, limiting the ability to establish causal relationships between sociodemographic factors and caregiver distress. Longitudinal studies are warranted to examine the temporal dynamics and potential bidirectional relationships between sociodemographic factors and caregiver distress over time. Directions for future research include conducting longitudinal studies to establish causal relationships between caregiving and psychological distress and investigating the impact of the intensity and duration of caregiving on psychological distress. Future studies should focus on information about the number of hours spent caregiving, the level of care required by the care recipient, and the duration of the caregiving role.

## Conclusions

This study highlights the significance of sociodemographic factors in understanding psychological distress among informal caregivers. Age, birth gender, household income, and education level emerged as key determinants of caregiver distress. By recognizing and addressing these sociodemographic factors, healthcare professionals and policymakers can develop targeted interventions to improve caregiver well-being and enhance the quality of care provided to vulnerable individuals.
